# The double-edged sword of nutraceuticals: comprehensive review of protective agents and their hidden risks

**DOI:** 10.3389/fnut.2025.1524627

**Published:** 2025-03-27

**Authors:** Sahand Ashrafpour, Manouchehr Ashrafpour

**Affiliations:** ^1^Student Research Committee, Babol University of Medical Sciences, Babol, Iran; ^2^Mobility Impairment Research Center, Health Research Institute, Babol University of Medical Sciences, Babol, Iran; ^3^Department of Physiology, School of Medicine, Babol University of Medical Sciences, Babol, Iran

**Keywords:** curcumin, resveratrol, piperine, quercetin, nutraceuticals, nutraceuticals-pharmaceutical interactions

## Abstract

Nutraceuticals-including resveratrol (RSV), curcumin (CUR), piperine (PPR), and quercetin (QUE)-exhibit dual therapeutic and toxicological profiles, are necessitating balanced risk–benefit evaluation. This review synthesizes evidence from about 120 preclinical/clinical studies sourced from PubMed, Scopus, and Web of Science using keywords (e.g., nutraceutical-drug interactions, bioavailability, CYP/P-gp modulation), prioritizing recent advances (2015–2024) alongside seminal works to contextualize mechanisms. Studies were selected based on methodological rigor, clinical relevance, and mechanistic insights into protective effects (antioxidant, anti-inflammatory, anticancer) and risks (organ toxicity, pro-oxidant activity, drug interactions). Key findings highlight PPR’s bioavailability-enhancing and neuroprotective properties, yet its inhibition of CYP3A4/P-gp elevates toxicity risks for carbamazepine (68.7% ↑ plasma concentration) and warfarin. CUR demonstrates hepatoprotective benefits but alters cardiovascular drug pharmacokinetics (e.g., amlodipine) and induces oxidative stress at high doses. RSV and QUE improve cardiovascular/neurological outcomes but interact with chemotherapeutics (RSV ↓ drug resistance via apoptosis; QUE ↑ methotrexate efficacy via anti-inflammatory synergy). Critical risks include reproductive toxicity (PPR >10 mg/kg), neurocognitive deficits (high-dose CUR), and CYP3A4-mediated interactions (QUE + cyclosporine). Nanotechnology-driven formulations (e.g., CUR/PPR nanoemulsions) mitigate risks by enhancing stability and enabling targeted delivery, though rigorous safety validation remains essential. This review underscores the need for evidence-based guidelines to optimize nutraceutical use in polypharmacy populations, emphasizing interdisciplinary collaboration to manage interactions. Innovations like nanoencapsulation could transition nutraceuticals from supplements to precision medicine adjuvants, pending resolution of dose–response ambiguities and long-term safety gaps through targeted research.

## Graphical abstract

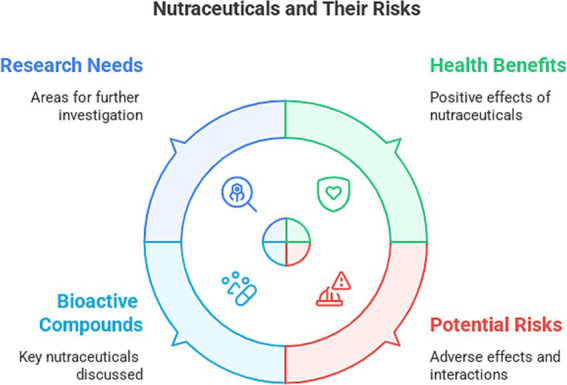

## 1 Introduction

The concept of using food as a form of medicine dates back thousands of years, when plants, herbs, and various foods were employed to treat illnesses and promote healing. Even in modern times, it is widely recognized that diet plays a significant role in influencing human physiology and overall health (1). Nutraceuticals, a term derived from “nutrition” and “pharmaceutical,” refer to food-derived products that provide health benefits beyond basic nutritional value. These bioactive compounds, available as dietary supplements, functional foods, and fortified products, have gained global popularity due to their natural origins and lower side-effect profiles compared to synthetic drugs (2). However, their therapeutic potential is accompanied by potential risks, necessitating a balanced understanding of their benefits and limitations.

Nutraceuticals are distinct from dietary supplements, which primarily provide essential nutrients like vitamins, minerals, and amino acids to address dietary deficiencies (3). In contrast, nutraceuticals, such as resveratrol (RSV), curcumin (CUR), piperine (PPR), and quercetin (QUE), are bioactive compounds with therapeutic properties that can modulate cellular pathways and disease processes at a biochemical level (4). These compounds are increasingly consumed to prevent or treat specific health conditions, owing to their antioxidant, anti-inflammatory, and neuroprotective effects. However, their pharmacokinetic profiles, including bioavailability and potential toxicity, vary significantly and require careful consideration.

**Figure 1:**
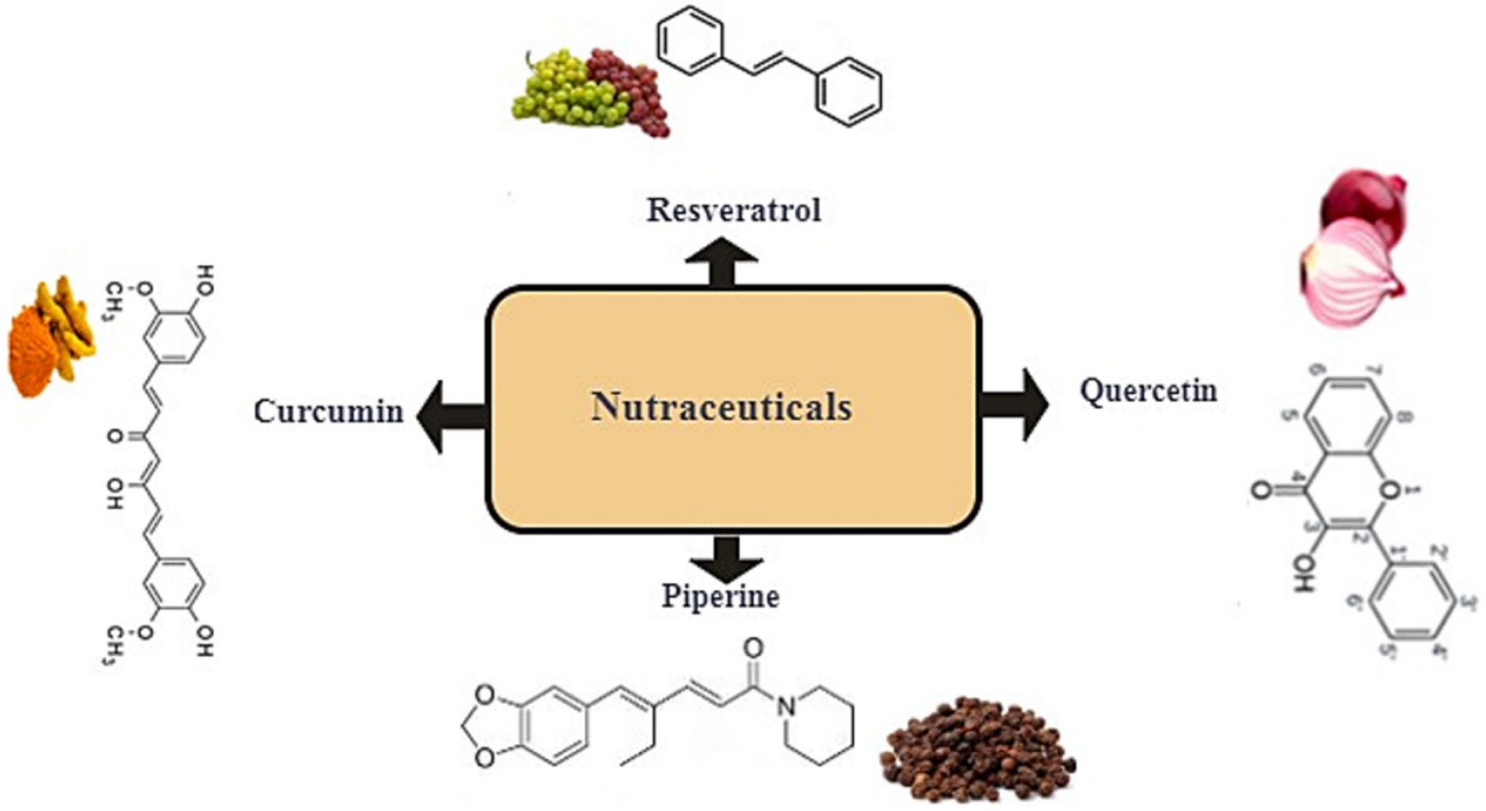
Representative classification of well-known nutraceuticals: resveratrol, curcumin, piperine, and quercetin.

The growing interest in nutraceuticals stems from their potential to address chronic diseases, such as cardiovascular disorders, neurodegenerative conditions, and metabolic syndromes, which are often linked to oxidative stress and inflammation (5, 6). For instance, four well-known nutraceuticals-RSV, CUR, PPR, and QUE-have been extensively studied for their protective effects on human health (Figure 1). These compounds demonstrate a wide range of potential beneficial effects, including antioxidant, anti-inflammatory, pro-apoptotic, and metabolic-regulating properties, which positions them as promising candidates for disease prevention and management. These compounds exhibit antioxidant, anti-inflammatory, and metabolic-regulating properties, making them promising candidates for disease prevention and management. However, despite their therapeutic potential, the safety and long-term efficacy of nutraceuticals remain understudied, particularly regarding dosage-specific toxicity, bioavailability, and interactions with pharmaceuticals.

While the protective effects of nutraceuticals are well-documented, their potential risks are often overlooked. Excessive consumption, prolonged use, or interactions with medications can lead to adverse outcomes, highlighting the need for a cautious approach to their use (7). For example, CUR, known for its hepatoprotective effects at low doses, may exhibit adverse effects at higher doses (8). Similarly, the bioavailability of these compounds, which is influenced by their formulation and delivery systems, remains a critical challenge that limits their therapeutic efficacy. Furthermore, the global regulatory landscape for nutraceuticals is inconsistent, complicating efforts to ensure their safety and quality (9).

The transition from traditional use to modern application underscores the enduring significance of bioactive compounds in human health. However, the complexity of their mechanisms and the lack of comprehensive studies on their long-term safety and efficacy necessitate further research. The primary purpose of this study is to provide a balanced and comprehensive review of the dual nature of nutraceuticals, with a focus on four major bioactive compounds. While these compounds are celebrated for their antioxidant, anti-inflammatory, and disease-preventive properties, their potential toxicities and interactions with pharmaceuticals are often overlooked. This review aims to:

1. Explore the mechanisms underlying their protective and potential challenges, including pro-oxidant activity, organ-specific alteration, and drug interactions.
2. Provide evidence-based recommendations for their safe and effective use, particularly in vulnerable populations and clinical settings.

By addressing these challenges, we hope to guide researchers, clinicians, and policymakers in making informed decisions about the use of nutraceuticals in health and disease management. This review is particularly timely given the growing global consumption of nutraceuticals and the increasing reports of adverse effects associated with their use. Addressing these potential risks is essential to maximize QUE’s therapeutic potential while minimizing its risks.

## 2 Beyond nutrition: the therapeutic and protective potential of nutraceuticals

In terms of mechanisms, nutraceuticals function as ligands that bind to various receptors, influencing critical cellular signaling pathways. This interaction directly affects processes such as neurogenesis, synaptogenesis, synaptic transmission, neuro-inflammation, oxidative stress management, cell death modulation, survival promotion, and structural remodeling of cells (9). For instance, RSV activates sirtuins-a family of proteins instrumental in regulating aging and inflammatory processes (10).

Moreover, another crucial aspect of their action lies in their antioxidant properties, which play a pivotal role in neutralizing free radicals and reactive oxygen species (ROS). Free radicals, being highly reactive molecules, can induce oxidative stress and damage vital cellular components like DNA, proteins, and lipids. Bioactive compounds such as polyphenols and flavonoids serve as effective scavengers of these free radicals, thereby reducing oxidative stress and safeguarding cellular integrity (7).

In addition to their potent antioxidant activities, these nutraceuticals also provide significant health benefits through their anti-inflammatory properties. The bioactive compounds within them can regulate inflammatory pathways by suppressing the production of pro-inflammatory cytokines and enzymes, such as tumor necrosis factor-alpha (TNF-*α*), interleukin-6 (IL-6), and cyclooxygenase-2 (COX-2) (11, 12). By reducing chronic inflammation, a key risk factor for various diseases-including cardiovascular disease, cancer, and neurodegenerative disorders-these nutraceuticals play a vital role in disease prevention and management (13–18).

Building on the previously discussed mechanisms by which bioactive food ingredients modulate inflammatory pathways, these compounds also play a crucial role in influencing metabolic processes. Specifically, bioactive food ingredients can activate or inhibit key enzymes and signaling pathways involved in metabolism, leading to improvements in lipid profiles, enhanced glucose metabolism, and better overall energy balance (19–21). This dual action—addressing both inflammation and metabolism-highlights their potential as natural therapeutic agents for promoting health and preventing chronic diseases.

Building on the mechanisms underlying nutraceuticals’ health-promoting and protective effects, the following section examines the distinct attributes of four key compounds-RSV, CUR, PPR, and QUE-whose unique properties underpin their efficacy in mitigating disease risk and enhancing therapeutic outcomes.

### 2.1 The therapeutic and protective potential of resveratrol

RSV, a polyphenolic compound abundant in grapes, red wine, and berries, has received significant scientific interest due to its wide-ranging therapeutic potential. Its health benefits-spanning cardiovascular, neurological, metabolic, and anti-inflammatory domains-are primarily mediated through antioxidant, anti-inflammatory, pro-apoptotic, and signaling pathway modulation mechanisms, as summarized in Table 1.

**Table 1:** Therapeutic and protective effects of resveratrol, curcumin, piperine, and quercetin.

Nutraceutical	Key beneficial effects	Mechanisms	Key references
Resveratrol	Cardioprotective, neuroprotective, anti-cancer, anti-inflammatory, antioxidant, metabolic benefits	Enhances endothelial function and reduces atherosclerosis. Reduces amyloid-beta accumulation in Alzheimer’s disease. Modulating lipid profiles. Activates sirtuins, promoting longevity.	(13–15, 22, 23)
Curcumin	Anti-inflammatory, antioxidant, neuroprotective, anti-cancer, metabolic benefits	Inhibits NF-κB and COX-2 pathways, reducing inflammation. Protects liver from oxidative damage. Induces apoptosis in cancer cells. Scavenges free radicals. Inhibits NF-κB. Activates PPARs, improves insulin sensitivity.	(11, 12, 20, 27–29)
Piperine	Bioavailability enhancer, neuroprotective, anti-inflammatory, antioxidant, metabolic benefits	Inhibits drug-metabolizing enzymes, enhancing bioavailability of other compounds. Reduces oxidative stress in the brain. Improves cognitive function and synaptic plasticity. Activates AMPK, regulates lipid metabolism.	(21, 32, 35–37, 39)
Quercetin	Antioxidant, anti-inflammatory, cardiovascular protection, neuroprotection, cardiovascular protection, metabolic and immune benefits	Scavenges free radicals and reduces oxidative stress. Improves endothelial function and reduces LDL cholesterol. Protects neurons from oxidative damage and inflammation. Enhances insulin sensitivity, modulates immune response.	(18, 43–48)

Cardiovascular Benefits: RSV exerts cardioprotective effects by enhancing endothelial function through increased nitric oxide (NO) production and reduced oxidative stress, promoting vasodilation and lowering blood pressure (13, 14). It also improves lipid metabolism by reducing LDL cholesterol and elevating HDL cholesterol, thereby mitigating atherosclerosis risk (22). These findings are supported by clinical trials, such as a randomized controlled study demonstrating that RSV supplementation significantly improves endothelial function and reduces hypertension (14), underscoring its role as a promising therapeutic agent for cardiovascular diseases.

Neuroprotective Potential: Beyond cardiovascular health, RSV shows remarkable neuroprotective efficacy. It inhibits amyloid-beta plaque accumulation in Alzheimer’s disease (AD) and enhances cognitive function by activating sirtuins (e.g., SIRT1) and modulating AMPK/NF-κB pathways (23). Clinical trials corroborate these effects: long-term, low-dose RSV supplementation improves memory, attention, and cerebrovascular function in postmenopausal women (15), underscoring its potential in combating neurodegenerative disorders like AD and Parkinson’s disease.

Anti-Cancer and Anti-Inflammatory Actions: RSV’s anti-cancer properties stem from its ability to regulate pathways governing cell proliferation, apoptosis, and angiogenesis. It selectively induces apoptosis in cancer cells while suppressing metastasis (7, 22). Concurrently, its potent anti-inflammatory effects mitigate chronic conditions such as rheumatoid arthritis and inflammatory bowel disease by inhibiting pro-inflammatory cytokines (e.g., TNF-*α*, IL-6) and enzymes like COX-2 (24). Notably, RSV reduces renal cell apoptosis by suppressing mitochondrial cytochrome C release and ROS levels, thereby alleviating oxidative damage (22).

Metabolic and Broader Health Implications: Emerging evidence highlights RSV’s metabolic benefits, including enhanced insulin sensitivity and glucose regulation via AMPK and sirtuin activation (19, 22). These mechanisms position RSV as a viable candidate for managing metabolic syndrome and type 2 diabetes.

Collectively, RSV’s pleiotropic effects underscore its potential as both a therapeutic adjunct for chronic diseases and a preventive agent in public health strategies. Further clinical research is warranted to optimize dosing regimens and validate long-term efficacy.

### 2.2 The therapeutic and protective potential of curcumin

CUR, the primary bioactive compound in turmeric (*Curcuma longa*), is widely consumed as a culinary spice and traditional remedy (11). Upon ingestion, it undergoes extensive hepatic and intestinal metabolism, yielding bioactive metabolites such as tetrahydrocurcumin, hexahydrocurcumin, and octahydrocurcumin, which retain biological activities comparable to the parent compound (25). Renowned for its antioxidant, anti-inflammatory, anti-cancer, and neuroprotective properties, CUR addresses diverse mechanisms underlying chronic diseases, as summarized in Table 1.

CUR’s antioxidant and anti-inflammatory effects form the cornerstone of its therapeutic profile. It scavenges free radicals, mitigates oxidative stress, and suppresses pro-inflammatory mediators like TNF-*α*, IL-6, and COX-2 by inhibiting the NF-κB signaling pathway (11, 12, 26, 27). These dual actions make it effective in managing conditions such as cardiovascular disease, diabetes, and arthritis.

Anti-Cancer Potential: CUR demonstrates significant anti-cancer properties by targeting multiple pathways involved in tumor progression. It inhibits tumor growth, induces apoptosis in cancer cells, and suppresses angiogenesis, thereby preventing the spread of cancer (28). These mechanisms have been observed in various cancers, including breast, prostate, and colorectal cancer, highlighting CUR’s potential as an adjunct to conventional cancer therapies.

Beyond oncology, CUR’s neuroprotective effects are notable. It reduces amyloid-beta plaques in Alzheimer’s disease, modulates neuroinflammation, and enhances cognitive function by crossing the blood–brain barrier (29). Clinical evidence highlights its ability to improve cognitive performance and lower amyloid-beta levels in older adults with mild cognitive impairment (30).

Emerging research further underscores CUR’s metabolic benefits, including enhanced insulin sensitivity and glucose regulation. By activating PPARs, it improves lipid metabolism and energy homeostasis, offering therapeutic potential for type II diabetes and obesity (12, 20).

Clinical validation reinforces these findings. A randomized controlled trial in patients with metabolic syndrome reported improved endothelial function and reduced inflammation following CUR supplementation (31). Collectively, CUR’s multifaceted properties-spanning antioxidant, anti-inflammatory, anti-cancer, neuroprotective, and metabolic domains-highlight its versatility in chronic disease management and preventive health strategies.

### 2.3 The therapeutic and protective potential of piperine

**Figure 2:**
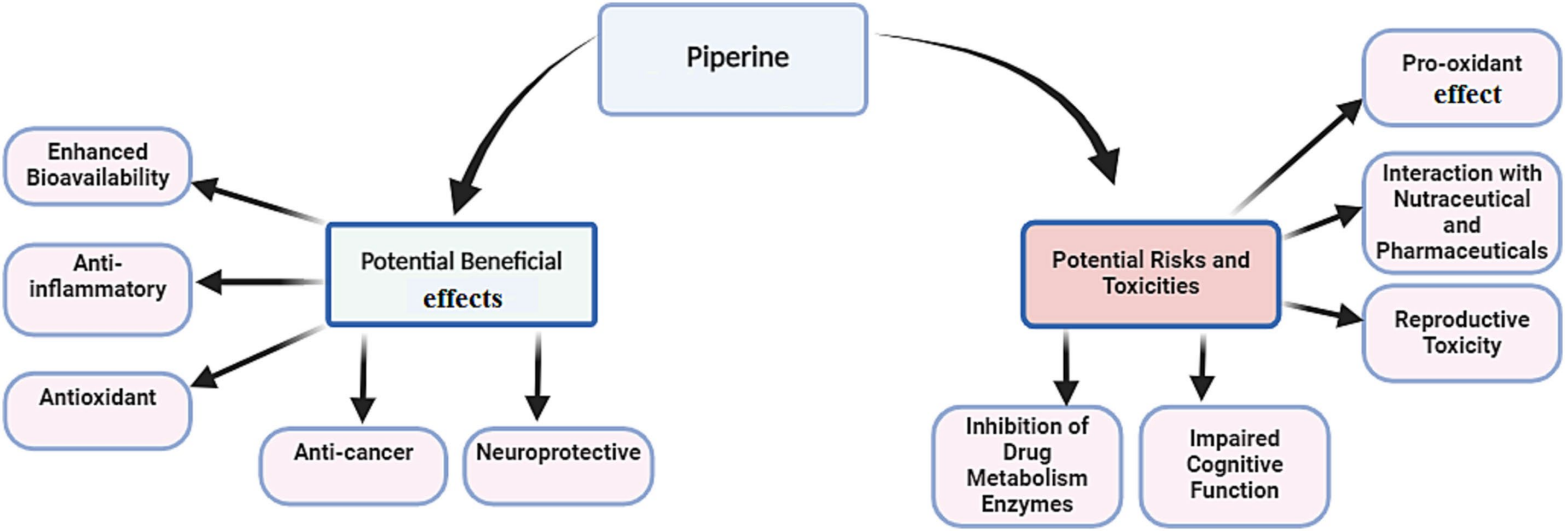
Potential beneficial effects of piperine alongside its associated risks and potential toxicities.

PPR, the bioactive alkaloid in black pepper (*Piper nigrum*), undergoes extensive metabolism in enterocytes and the liver, where glucuronidation, sulfation, and methylation produce water-soluble derivatives for excretion (27). As illustrated in Table 1 and Figure 2, PPR is well-known for its diverse health benefits. Its therapeutic potential lies in its ability to enhance bioavailability, reduce inflammation, combat oxidative stress, inhibit cancer progression, and protect neural function. These properties collectively address key mechanisms involved in chronic diseases.

Bioavailability Enhancement: A hallmark of PPR is its ability to augment the bioavailability of drugs and nutrients. By inhibiting drug-metabolizing enzymes (e.g., cytochrome P450, UDP-glucuronosyltransferase) and enhancing intestinal absorption (21, 32), PPR significantly inhibits P-glycoprotein (P-gp) and enhances the efficacy of co-administered compounds like CUR and RSV, positioning it as a critical adjunct in nutraceutical formulations (33, 34).

Building on this foundational role, PPR’s antioxidant effects further contribute to its therapeutic profile. It reduces oxidative stress by scavenging free radicals (35, 36) and suppresses inflammatory and pro-inflammatory cytokines (e.g., TNF-*α*, IL-6) (37), offering promise in managing chronic inflammatory conditions such as arthritis and metabolic disorders.

Expanding into oncology, PPR demonstrates anti-cancer properties by inhibiting cancer cell proliferation and inducing apoptosis in breast, colon, and prostate cancers (38, 39). Its modulation of key pathways like PI3K/AKT and NF-κB disrupts tumor growth and metastasis, underscoring its potential as an adjunct to conventional therapies.

In neuroprotection, PPR improves cognitive function and mitigates neurodegenerative pathology. By modulating neurotransmitter levels, reducing neuroinflammation (40), and enhancing cognitive function through oxidative stress protection and increased hippocampal synaptic plasticity (36), it exerts protective effects against AD, as evidenced in preclinical studies.

Emerging research highlights PPR’s metabolic benefits, including enhanced insulin sensitivity and lipid metabolism regulation via AMPK activation and adipocyte differentiation modulation (21). These mechanisms position PPR as a candidate for managing obesity and type II diabetes.

### 2.4 The therapeutic and protective potential of quercetin

**Figure 3:**
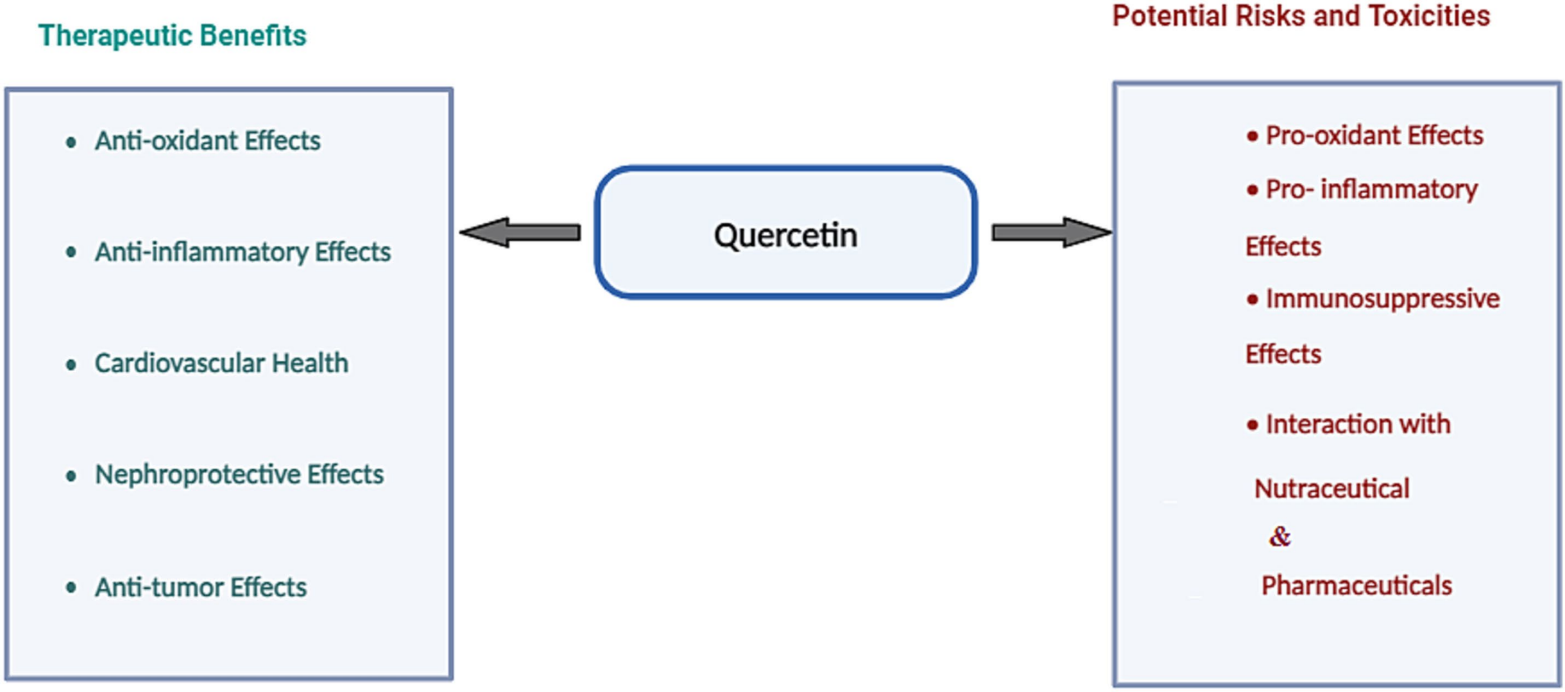
Potential beneficial effects of quercetin alongside its associated risks and potential toxicities.

QUE, a flavonoid abundant in fruits and vegetables, has emerged as a potent nutraceutical with antioxidant, anti-inflammatory, cardiovascular, and neuroprotective properties, positioning it as a versatile agent for chronic disease prevention and management (Table 1; Figure 3). While QUE glycosides—bound to sugar residues—are commonly consumed through dietary sources like apples and onions (41), its aglycone form is predominant in supplements, offering enhanced bioavailability (42).

Antioxidant Effects: QUE’s hallmark lies in its ability to neutralize free radicals and mitigate oxidative stress, a key driver of chronic conditions such as cancer and cardiovascular disease (43). By scavenging ROS and bolstering endogenous antioxidant defenses, it safeguards cellular integrity, protecting DNA, proteins, and lipids from oxidative damage.

Building on its antioxidant prowess, QER exerts anti-inflammatory effects through inhibition of pro-inflammatory cytokines (e.g., TNF-*α*, IL-6) and enzymes like COX-2, mediated via NF-κB and MAPK pathway modulation (44, 45). These properties are exemplified in a study by Demkovych et al. (46), where water-soluble QUE (corvitin) stabilized oxidative balance in rats with bacterial-immune periodontitis by reducing superoxide dismutase activity and sustaining catalase and ceruloplasmin levels. Such findings underscore its therapeutic potential in inflammatory disorders like arthritis and inflammatory bowel disease.

Expanding to cardiovascular health, QUE enhances endothelial function, lowers blood pressure, and reduces LDL cholesterol, thereby curbing atherosclerosis risk (16, 47). Its antioxidant capacity further prevents LDL oxidation, a critical factor in cardiovascular disease progression.

In neuroprotection, QUE demonstrates promise in combating neurodegenerative diseases such as Alzheimer’s and Parkinson’s. By inhibiting beta-amyloid plaque formation, reducing neuronal oxidative damage, and dampening neuroinflammation (17, 18), it offers a multifaceted approach to brain health.

Emerging research highlights QUE’s metabolic and immune benefits, including improved insulin sensitivity and glucose metabolism regulation, vital for managing metabolic disorders (48). Concurrently, its immunomodulatory properties make it a candidate for addressing autoimmune and inflammatory conditions, bridging metabolic and immune health.

Overall, QUE’s multifaceted actions position it as a cornerstone of preventive and therapeutic strategies. Its synergy with pharmaceuticals and nutraceuticals further cements its role in bridging nutrition and pharmacotherapy, offering holistic solutions for complex health challenges.

## 3 Understanding nutraceutical risks and challenges

However, despite their widespread use and assumed safety, emerging evidence challenges this assumption, revealing potential risks associated with nutraceuticals-particularly at high doses, during prolonged use, or in combination with other bioactive compounds. These risks arise through direct or indirect interactions with cellular systems, which may disrupt homeostasis, trigger oxidative stress, and dysregulate critical molecular pathways that can culminate in organ toxicity or metabolic dysfunction. In the United States, dietary supplements account for approximately 20% of drug-induced liver injuries, with nearly half attributed to combination products (49). Similarly, a Spanish registry documented herbal products as the cause of 2% of drug-induced liver injury cases (50), highlighting the need to evaluate nutraceutical safety through the lens of cellular and molecular interactions.

Antioxidants, typically known for neutralizing harmful ROS, can also act as prooxidants under certain conditions. In the presence of reduced metals (iron, copper), antioxidants like flavonoids can generate toxic ROS instead of protecting cells. This dual behavior is influenced by factors such as dose, environmental conditions, and the presence of metal ions. For instance, high doses of antioxidants like beta-carotene or vitamin E may not always be beneficial and could even exacerbate oxidative damage, particularly before exposure to harmful agents like radiation or cigarette smoke (51).

Many bioactive compounds, including flavonoids, exhibit dose-dependent pro-oxidant activity. At low concentrations, they function as antioxidants, but at higher doses, they can promote the formation of free radicals, leading to oxidative stress and cellular damage. At high concentrations, QUE catalyzes the reduction of metal ions, generating ROS and causing oxidative damage to DNA and other cellular components (52). Similarly, PPR, due to the presence of amide groups in its structure, can exhibit pro-oxidant activity under certain conditions, increasing ROS production and oxidative stress (53). In the following sections, we will explore how some of these nutraceutical agents, despite their well-documented antioxidant properties, can potentially act as pro-oxidants under specific conditions.

### 3.1 Resveratrol: potential risks and challenges

RSV is among the most extensively studied natural polyphenols, well-known for its numerous health benefits, including antioxidant, anti-inflammatory, and cardioprotective effects. However, findings from human clinical trials have yielded mixed results regarding its protective effects against various diseases and their associated complications (Table 2). While RSV is generally considered safe for human consumption, emerging evidence suggests that it may exhibit toxic effects under certain conditions, particularly at high doses or with prolonged use (24). However, the toxicity of RSV remains subject to controversy, with conflicting evidence across studies.

**Table 2:** Potential adverse effects and toxicity profiles of resveratrol, curcumin, piperine, quercetin.

Nutraceutical	Organ-specific adverse effects	Pro-oxidant effects	Drug interactions	Reproductive toxicity	Dose-dependent effects	Key references
Resveratrol	Chemically-induced organ injury concern	High doses increase oxidative stress and DNA damage	Inhibits cytochrome P450 enzymes	Limited evidence, but potential hormonal disruption	Low doses: antioxidant; high doses: pro-oxidant	(7, 24, 69–71)
Curcumin	Gastrointestinal irritation, potential tissue-damaging effect	High doses cause DNA damage and oxidative stress	Inhibits CYP3A4, alters drug bioavailability	Reduces sperm motility and fertility	Low doses: anti-inflammatory; high doses: toxic	(72–80)
Piperine	Organ-specific adverse effect, drug negative interaction	Limited pro-oxidant effects	Broad-spectrum inhibitor of drug metabolism	Reduces sperm count, motility, and viability	Low doses: protective; high doses: toxic	(40, 81–86)
Quercetin	Potential metabolic and tissue damaging concern	High doses induce oxidative stress and ROS	Inhibits drug-metabolizing enzymes and transporters	Limited evidence, but potential reproductive effects	Low doses: antioxidant; high doses: pro-oxidant	(42, 73, 88–92)

One of the primary concerns with RSV is its dose-dependent biphasic and hormetic effects. At low concentrations, RSV functions as an antioxidant by directly scavenging ROS and downregulation NADPH oxidases, thereby protecting against DNA damage and oxidative stress. Conversely, at elevated concentrations, RSV acts as a pro-oxidant, upregulating NADPH oxidases and increasing oxidative stress, which can lead to DNA damage and cellular senescence (7). This dual behavior is influenced by factors such as concentration, treatment duration, cell type, and the basal redox state of the cells.

RSV’s potential to influence the bioavailability and therapeutic efficacy of other drugs is another critical concern. At high doses, RSV can inhibit cytochrome P450 (CYP3A4) enzymes (54), altering the metabolism of co-administered drugs and potentially leading to adverse effects. Additionally, long-term consumption of RSV has been associated with goitrogenic properties, which may disrupt thyroid function (24, 55). The timing of RSV administration also plays a significant role in its effects. Chronobiological studies have shown that RSV decreases lipid peroxidation when administered during the dark phase but increases it during the light phase, highlighting the importance of timing in its therapeutic application (24). The results of a study showed that, in both *in vitro* and *in vivo* conditions, rats receiving RSV with daily dose of 25 mg/kg for 60 days exhibited elevated TSH levels. These findings were further confirmed by immunohistochemical analysis (55).

Clinical studies have further underscored the potential risks of RSV. For example, Popat et al. (56) reported that administering a high dose of RSV (5 g/day) to multiple myeloma patients unexpectedly caused renal damage in five cases, leading to the premature termination of the trial. Similarly, animal studies have demonstrated that while low doses of RSV promote the healing of indomethacin-induced gastric ulcers, higher doses exacerbate ulcer severity and delay the healing of pre-existing ulcers (57). These findings suggest that RSV has a narrow therapeutic window, with beneficial effects at low doses and potential adverse effects at high doses.

### 3.2 Curcumin: potential risks and challenges

Despite numerous studies highlighting the potential benefits of CUR, certain adverse effects and toxicities have been reported, particularly at high doses or with prolonged use. While CUR longa and its active compound CUR are generally well-tolerated, emerging evidence suggests potential risks that remain under debate. Some of the potential risks and challenges associated with CUR are outlined in Table 2.

One of the most commonly reported adverse effects of CUR is gastrointestinal irritation, which can manifest as nausea, diarrhea, and abdominal pain, particularly at high doses (58, 59). These symptoms are often dose-dependent and may limit the tolerability of CUR in some individuals.

Alongside the hepatoprotective effects of CUR, which have been demonstrated in the literature, instances of liver alteration associated with its use have also been reported (58, 60). There are also reports regarding its impact on thyroid function. For instance, a study by Papiez et al. (61) demonstrated that CUR administration significantly increased free plasma T3 and T4 levels in young rats but decreased free serum T3 levels in older rats, suggesting age-dependent effects on thyroid function. However, the extent to which these adverse effects can be directly attributed to CUR remains unclear.

CUR’s pro-oxidant effects at high doses have also raised concerns about its potential to cause DNA damage and cellular toxicity. Animal studies have shown that high-dose CUR, particularly when delivered via nanoparticles, can induce tissue toxicity through inflammation-mediated injury (62, 63). These findings suggest that while CUR has therapeutic potential, its dose-dependent dual nature—acting as both an antioxidant and a pro-oxidant—requires careful consideration to avoid unintended consequences.

The potential challenges associated with CUR further complicate its therapeutic application. For instance, a study by Cianfruglia et al. (64) showed that CUR concentrations exceeding 10 μM induced cell death in normal human dermal fibroblast cells. This finding highlights concerns regarding its possible adverse effects on healthy tissues, underscoring the need for careful dose optimization and further investigation into its safety profile.

According to animal experiments, CUR has been shown to inhibit sperm motility and function in a concentration-dependent manner, significantly reducing fertility in both *in vitro* and *in vivo* models (65, 66). These findings highlight its “double-edged sword” status and the importance of cautious dosing and thorough safety assessments in preclinical and clinical settings.

### 3.3 Piperine: potential risks and challenges

PPR is widely recognized for its ability to enhance the bioavailability of other nutraceuticals and pharmaceuticals. However, despite its long history of use and perceived safety, emerging evidence suggests that PPR may pose significant risks, particularly at high doses or with prolonged use. These risks include organ-specific changes, reproductive toxicity, and drug interactions, underscoring the need for cautious use and further research. Some of the potential risks and challenges associated with PPR are outlined in Table 2 and Figure 2.

One of the primary concerns with PPR is its metabolic transformation into potentially harmful metabolites. Several metabolites of PPR are formed through processes such as hydrogenation and modification of the methylenedioxyphenyl ring, which can alter its biological activity and raise toxicity concerns (40, 67, 68). These structural modifications may lead to adverse effects, highlighting the importance of understanding PPR’s metabolic pathways and their implications for safety.

Animal studies have provided mixed evidence regarding PPR’s toxicity. While low doses of PPR have been associated with neuroprotective effects, such as enhanced cognitive function and prevention of oxidative stress in the brain, high doses have been shown to impair cognitive performance and dampen long-term potentiation (69). Additionally, findings from a recent study indicate that high doses of PPR may lead to cardiac adverse effects in rat (unpublished data), further complicating its safety profile.

PPR’s impact on male reproductive health is another area of concern. Studies have shown that high doses of PPR (e.g., 10 mg/kg) significantly reduce sperm motility, concentration, and viability in adult male rats, with adverse effects on key reproductive markers and antioxidant enzymes (70, 71). Although many of these effects were reversible after a withdrawal period, they highlight the potential risks of prolonged or high-dose PPR use.

A recent study examining the sub chronic effects of PPR in mice identified significant alterations in the histopathological characteristics of certain internal tissues. At doses as low as 35 mg/kg, notable changes were observed, potentially affecting physiological function. Higher doses (140 mg/kg) further exacerbated these effects, leading to more pronounced tissue modifications that could influence overall health outcomes (72). These findings highlight the importance of careful dose selection when evaluating PPR’s safety and efficacy.

Despite its broad therapeutic potential, PPR’s clinical applications are limited by its dose-dependent toxicity and potential interactions with pharmaceuticals. Recent advancements in delivery systems, such as nanotechnology-based formulations, have shown promise in optimizing PPR’s therapeutic efficacy while minimizing adverse effects (73). These innovations have significantly improved PPR’s stability and bioavailability, paving the way for more effective therapeutic applications.

### 3.4 Quercetin: potential risks and challenges

QUE, a widely studied flavonoid, is distinguished for its antioxidant, anti-inflammatory, and neuroprotective properties. However, emerging evidence suggests that QUE may also pose significant risks, particularly at high doses or under specific conditions. Some of the potential risks and challenges associated with QUE are outlined in Table 2 and Figure 3.

QUE undergoes extensive metabolic transformation in the body, primarily in the enterocytes and liver, leading to the formation of numerous metabolites. Some of these metabolites, such as QUE –quinone methide, have the potential to cause cellular or tissue damage due to their pro-oxidant properties. These metabolites can act as electrophiles and contribute to the production of ROS through redox cycling mechanisms (42, 74, 75), highlighting the dual nature of QUE’s biological effects. Animal studies have further demonstrated QUE’s dose-dependent changes in the body. For example, research using zebrafish models revealed that while low doses of QUE provide antioxidant benefits, high doses induce oxidative stress and cause hepatic and renal alterations (59). Similarly, QUE –Cu (II) complexes can generate harmful hydroxyl radicals through Fenton-like reactions, highlighting its pro-oxidant potential under certain conditions (76). These findings emphasize the need to balance QUE’s therapeutic benefits with its potential risks.

The dose-dependent dual nature of QUE is a key concern. At low doses, QUE exhibits antioxidant and anti-inflammatory effects, as demonstrated in a study where a low dose (10 mg/kg) reduced pro-inflammatory markers (TNF-*α* and IL-1β) in rats with doxorubicin-induced nephrotoxicity. However, at higher doses (100 mg/kg), QUE increased oxidative stress markers and reduced key antioxidants (e.g., glutathione and catalase), exacerbating renal damage (77). In addition, it has been reported that QUE, at high doses, induces oxidative stress, leading to neuronal damage and exacerbation of neurodegenerative conditions in experimental *in vitro* models of neurodegeneration (78). This paradoxical effect underscores the importance of careful dosing in therapeutic applications.

In summary, QUE’s dual, dose-dependent nature—characterized by protective effects at low doses and toxic effects at high doses—highlights its “double-edged sword” status. While it offers significant promise as a therapeutic agent, its potential for pro-oxidant activity, organ-specific toxicity, and drug interactions necessitates careful consideration of dosing regimens and further research to establish safe and effective use.

## 4 Uncovering potential nutraceutical interactions: mechanisms, risks, and clinical implications

The study of nutraceutical interactions encompasses two key domains: nutraceutical-nutraceutical and nutraceutical-pharmaceutical interactions. The interplay between nutraceuticals and pharmaceuticals has emerged as a critical focus in clinical research, driven by the dual potential to enhance therapeutic outcomes or inadvertently compromise patient safety (79, 80). Nutraceuticals are increasingly being utilized to address age-related cognitive impairments due to their neuroprotective and cognitive-enhancing properties (9). However, their growing popularity as cognitive enhancers raises concerns, particularly among older adults who use multiple medications, as this can increase the risk of adverse interactions (81). A thorough understanding of the pharmacokinetic and pharmacodynamic profiles of these compounds is essential for predicting potential adverse drug reactions and preventing therapeutic failure.

### 4.1 Nutraceutical-pharmaceutical interactions

International epidemiological studies highlight significant variability in the global incidence of interactions between nutritional supplements and pharmacological agents, with reported rates ranging from 6 to 70% (82). These interactions are particularly prevalent among elderly individuals, patients with chronic conditions, and populations undergoing polypharmacy regimens (81). A key contributor to this variability is the role of metabolic enzymes such as CYP3A4, which processes approximately 70% of clinically used medications. Notably, nutraceuticals like RSV, PPR, and QUE have been shown to inhibit CYP3A4 activity, thereby reducing systemic drug elimination rates and increasing toxicity risks due to prolonged drug retention in circulation (9, 21, 32, 54).

Furthermore, such interactions extend beyond CYP3A4. For example, PPR exhibits competitive/reversible and non-selective inhibition of monoamine oxidase (MAO) enzymes, which are critical targets for anti-Parkinsonian and antidepressant therapies like MAO inhibitors (83). Additionally, certain bioactive compounds induce CYP enzyme activity, accelerating drug metabolism and reducing therapeutic efficacy. St. John’s Wort and PPR, for example, induce CYP3A4 and P-gp, increasing the clearance of oral contraceptives, antivirals, and immunosuppressants (84). Similarly, CUR alters the pharmacokinetics of blood pressure medications, potentially compromising their efficacy or safety (80). This underscores the complexity of nutraceutical-drug interplay and its implications for clinical safety.

Emerging evidence highlights the dual role of nutraceuticals in modulating drug efficacy and toxicity. For instance, PPR significantly alters the pharmacokinetics of carbamazepine, increasing its maximum plasma concentration, half-life, and area under the curve by 68.7, 47.9, and 43.2%, respectively—elevating risks of drug toxicity (85). Preclinical studies further demonstrate that combining PPR with donepezil enhances therapeutic efficacy in AD rat models by improving synaptic plasticity, reducing oxidative stress, and ameliorating histopathological outcomes (36). These findings underscore the potential of nutraceutical synergies to optimize therapeutic outcomes while reducing pharmaceutical dosages and minimizing side effects.

Clinically, nutraceutical-pharmaceutical interactions are as critical as nutraceutical-nutraceutical interactions. For example, RSV enhances chemotherapeutic efficacy by promoting apoptosis and reducing drug resistance (86), while QUE amplifies methotrexate’s anti-inflammatory effects via dual targeting of oxidative stress and inflammation (45). QUE also shows promise in boosting chemotherapeutic efficacy, offering improved outcomes for cancer patients (87). However, such benefits are counterbalanced by risks: P-gp-mediated drug efflux, a key contributor to therapeutic resistance for drugs like MAO inhibitors and anticancer agents, can be modulated by nutraceuticals like PPR and QUE (81, 88). By inhibiting P-gp, these agents enhance cellular drug retention but may inadvertently increase systemic drug concentrations, posing toxicity risks for narrow-therapeutic-index medications (e.g., chemotherapeutics, cardiovascular drugs) (32, 88–90).

QUE exerts dose-dependent inhibition of drug-metabolizing enzymes and transporters in rats, significantly altering cyclosporine pharmacokinetics (89, 90). This underscores the need for close monitoring when combining QUE-containing supplements with drugs metabolized by P-gp or CYP3A4 (e.g., cyclosporine). Similarly, CUR inhibits CYP3A4 and induces P-gp, altering the bioavailability of calcium channel blockers (e.g., amlodipine) and beta-blockers (e.g., carvedilol) (80). Such interactions are particularly critical for narrow-therapeutic-index drugs like warfarin, digoxin, and flecainide, where minor dosage changes can trigger severe adverse effects (86). For example:

- PPR inhibits cytochrome P450 enzymes, reducing metabolism of warfarin’s active metabolite (7-hydroxywarfarin) and potentially diminishing anticoagulant efficacy (91).
- Conversely, co-administering CUR with warfarin increases its bioavailability, elevating bleeding risks (92).
- *In vitro* studies reveal that CUR alone up-regulates CYP3A (but not CYP1A2), while combined CUR/QUE and warfarin up-regulates both CYP3A and CYP1A2, altering warfarin metabolism (93).

While combining nutraceuticals with pharmaceuticals can amplify efficacy (e.g., enhancing drug absorption) or enable dose reduction (87, 89, 90), such synergies require meticulous scrutiny. Unmanaged interactions may disrupt drug metabolism pathways, heightening toxicity or reducing therapeutic effectiveness-particularly in vulnerable populations (49, 94). For instance, conflicting findings on PPR-warfarin interactions highlight this complexity: some studies report PPR-mediated inhibition of warfarin metabolism, increasing plasma concentrations and anticoagulant effects (95), while others suggest diminished efficacy (91).

**Figure 4:**
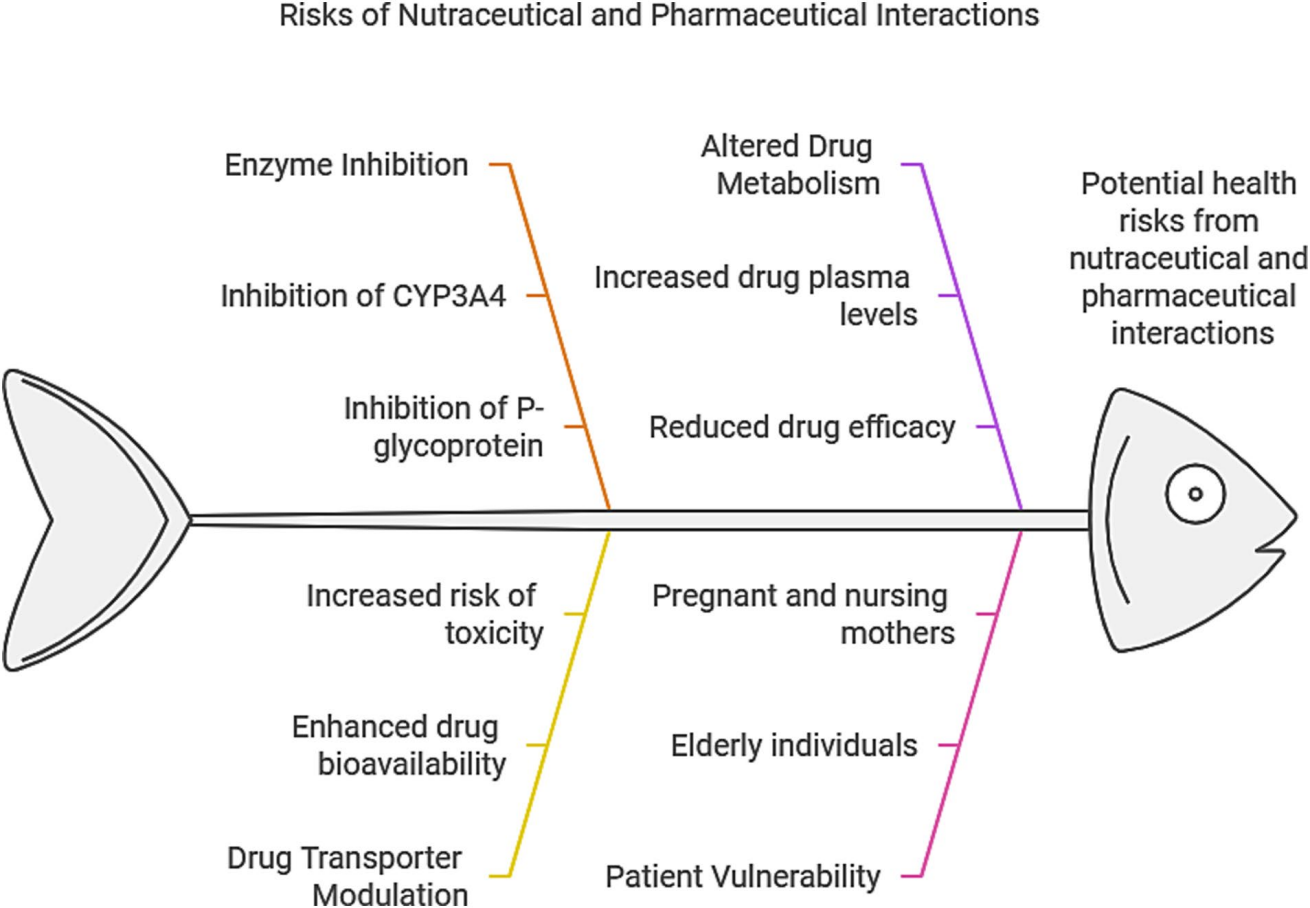
Potential interactions between nutraceuticals and pharmaceuticals.

These dual effects emphasize the need for evidence-based integration of nutraceutical-pharmaceutical regimens. A schematic overview of nutraceutical impacts on drug transport/metabolism, interaction mechanisms, and at-risk populations is provided in Figure 4. To mitigate risks, interdisciplinary collaboration among pharmacists, physicians, and nutrition specialists is essential to systematically identify and manage medication-nutrient interactions (96).

### 4.2 Nutraceutical-nutraceutical interactions

Emerging evidence highlights the therapeutic potential of synergistic nutraceutical combinations. For instance, co-administering CUR with PPR or RSV significantly enhances CUR’s bioavailability, amplifying its anti-inflammatory (33), apoptotic, and anticancer effects (34, 97). Similarly, combining RSV with PPR improves cerebral blood flow and oxygen utilization, thereby boosting therapeutic efficacy in neurological conditions (10). These findings underscore the capacity of strategic nutraceutical pairings to optimize pharmacological outcomes.

Despite their benefits, nutraceutical combinations may inadvertently increase toxicity risks. A notable example is the CUR-PPR interaction: while PPR enhances CUR’s bioavailability by inhibiting CYP3A4 and P-gp—critical enzymes for CUR metabolism—via competitive binding to their active sites, this inhibition prolongs systemic CUR exposure. Molecular docking studies reveal that hydrogen-bonded complexes formed between PPR and CUR further facilitate metabolic transport, raising toxicity concerns (32). For instance, adding 20 mg of PPR to turmeric increases CUR’s serum bioavailability by 20-fold (98), and co-administering PPR with RSV (10 mg/kg in mice) elevates RSV’s maximum plasma concentration by over 1,500% compared to solo administration at 100 mg/kg (10). Such interactions emphasize the need for cautious evaluation of bioavailability-enhancing strategies to balance efficacy and safety.

Recent advancements in nanotechnology-driven formulations, such as nanoencapsulation, offer promising solutions to mitigate risks while enhancing therapeutic outcomes. These systems improve the bioavailability and stability of nutraceuticals like PPR, enabling precise control over delivery and reducing toxicity risks (40). For instance, nanoparticle-based delivery optimizes PPR’s efficacy while minimizing adverse effects, highlighting the potential of engineered formulations to bridge safety and efficacy gaps.

Although nutraceuticals like CUR, PPR, and RSV are classified as Generally Recognized as Safe (GRAS) by the U.S. FDA for dietary use, none have received formal approval as pharmaceutical agents for disease treatment or prevention (99–101). Their GRAS status underscores the need for rigorous clinical trials to validate therapeutic efficacy, safety, and optimal dosing in medical contexts. Persistent knowledge gaps regarding interaction dynamics and long-term safety further necessitate evidence-based frameworks to guide their integration into clinical practice.

### 4.3 Clinical implications: balancing benefits and risks in practice

Healthcare providers should implement targeted strategies to address nutraceutical-related risks, focusing on comprehensive reviews of nutraceutical-drug interactions and the discontinuation of non-essential supplements in high-risk populations, such as the elderly or those undergoing polypharmacy (102). To facilitate safer recommendations, tools like the Natural Medicines Comprehensive Database or Drug Interaction Checkers can identify clinically significant nutraceutical-drug pairs. Additionally, collaborative care models involving pharmacists and nutrition experts are essential for reconciling nutraceutical use with prescribed medications, while patient education should emphasize the importance of disclosing all supplements during clinical consultations (103). Integrating nutraceutical documentation into electronic health records (EHRs) can further reduce the risk of adverse outcomes, aligning with emerging guidelines that advocate for proactive management of nutraceutical risks in polypharmacy care (104).

Furthermore, emerging tools such as in silico methodologies (105), and the Nutraceutical Interaction Risk Score (NIRS) (106), offer valuable resources for clinicians. These tools can predict potential toxicities and stratify patients into low-, moderate-, or high-risk categories based on factors like age, polypharmacy status, and metabolic enzyme polymorphisms. For high-risk patients, alternatives such as dietary modifications may provide safer synergies with conventional therapies, potentially enhancing treatment safety and efficacy (107).

The dual nature of nutraceuticals underscores the critical need for vigilant biomarker monitoring in clinical practice (93, 94). For example, patients who combine nutraceuticals with anticoagulants may experience clotting modifications due to alterations in CYP enzyme activity (93, 96), necessitating regular coagulation monitoring on a weekly basis to ensure safety and efficacy.

Nutraceuticals can influence not only pharmacodynamic interactions but also affect their absorption, metabolism, and excretion characteristics. Most studies related to nutraceutical-pharmaceutical interactions focus on their impact on CYP enzymes or P-gp transporters. However, these risks can also be exacerbated by CYP genetic polymorphisms and liver diseases, which may increase the production of potentially hepatotoxic drug metabolites (108, 109). Emerging tools, such as pharmacogenetic (110) and nutrigenomic (111) tests now facilitate large-scale pharmacogenomic and nutrigenomic analyses, enabling the early identification of genetic variants that affect nutraceutical-drug interactions. By translating genomic data into actionable insights, these tools play a crucial role in developing personalized monitoring strategies (112), particularly for elderly patients or those on polypharmacy regimens who use high-risk nutraceuticals. This approach not only improves biomarker monitoring but also equips clinicians to optimize the safety of nutraceutical use more effectively.

## 5 Nanotechnology in nutraceuticals: innovations for personalized therapies and enhanced bioavailability

Recent advancements in nanotechnology have revolutionized the field of nutraceuticals, offering innovative solutions to longstanding challenges such as poor solubility, instability, and low bioavailability of bioactive compounds. By leveraging nanoscience, researchers have developed sophisticated delivery systems-including liposomes, nanoemulsions, and polymeric nanoparticles-that encapsulate nutraceuticals, shielding them from degradation and enabling controlled, targeted release. For instance, nanoformulations of CUR and RSV have demonstrated significantly enhanced absorption rates and therapeutic efficacy against cancer cells compared to their conventional counterparts, underscoring the transformative potential of nanotechnology in nutraceutical delivery (10, 28, 113).

Interestingly, an *in vitro* study has demonstrated that nanoemulsified particles of CUR or PPR exhibit greater efficacy compared to their free forms when used in cancer cell lines (34). Specifically, while nanoemulsified CUR resulted in a 4-fold increase in Caspase 3 levels-a key marker of apoptosis-the combination treatment involving nanoemulsified PPR led to a 6-fold elevation of this marker. These findings underscore the substantial improvement in anticancer activity achieved through the use of nanoemulsified particles of PPR and CUR, highlighting the potential advantages of nanoemulsion-based delivery systems for nutraceuticals.

A key advantage of these systems lies in their ability to enable personalized therapies. By tailoring nanoparticle size, surface charge, and functionalization, nutraceuticals can be engineered to target specific tissues or respond to physiological triggers (e.g., pH or enzymatic activity), ensuring precision delivery. This approach aligns with the growing demand for individualized nutrition, particularly in managing chronic diseases such as diabetes and cardiovascular disorders (73, 74).

Furthermore, nanotechnology contributes to sustainability in nutraceutical development. As highlighted in “Innovations in nanoscience for the sustainable development of food and agriculture with implications on health and environment” (114), biodegradable and eco-friendly nanoformulations reduce environmental impact while maintaining efficacy. For example, lipid-based nanoparticles derived from natural sources minimize toxicity and align with green chemistry principles. However, the adoption of these technologies necessitates rigorous safety evaluations and harmonized regulatory frameworks to address concerns about long-term biocompatibility.

Looking ahead, the integration of artificial intelligence in nanocarriers design and the development of multifunctional “theranostic” systems-combining therapy and diagnostics-promise to unlock new frontiers in nutraceutical science. These innovations not only enhance therapeutic outcomes but also pave the way for sustainable, patient-centric healthcare solutions (115, 116).

From a prospective viewpoint, the exploration of novel therapies that are both effective and less toxic to normal cells represents a promising frontier in treatment development. Herbal compounds, well-known for their potent protective and anti-cancer properties, have emerged as potential candidates for smart medicine when formulated using nanotechnology-based approaches (117). The integration of nanotechnology into this field has further advanced the potential of these compounds by enabling the creation of smart nanomaterials that improve solubility, bioavailability, and metabolic stability (118). Furthermore, these innovations facilitate the controlled and targeted delivery of therapeutic agents directly to tumor sites or damaged organs, thereby minimizing off-target effects and systemic exposure (117, 118). This approach not only enhances the efficacy of herbal compounds but also significantly reduces toxicity, creating new opportunities for safer and more personalized treatment strategies.

On the other hand, while experimental approaches provide valuable insights into the biological effects of nutraceuticals, in silico molecular docking serves as a powerful computational tool to predict and elucidate the binding interactions between these compounds and their molecular targets (119). For instance, docking studies can reveal how bioactive compounds such as CUR or PPR interact with key inflammatory mediators (e.g., NF-κB, COX-2) or metabolic regulators (e.g., PPAR-*γ*, AMPK) (21, 120), offering mechanistic insights into their synergistic or antagonistic effects when combined with pharmaceuticals. These analyses not only complement experimental findings but also enhance our understanding by identifying potential binding affinities, binding sites, and conformational changes, thereby validating hypothesized mechanisms of action and guiding the development of more effective therapeutic strategies.

## 6 Limitations

While this study provides critical insights into the mechanisms and protective potential of RSV, CUR, PPR, and QUE nutraceuticals, several limitations warrant acknowledgment. First, our findings derive primarily from short-term trials and preclinical models, which may not fully recapitulate human physiological complexity, long-term safety outcomes, or inter-individual variability in clinical populations. Second, variability in nutraceutical formulations and dosing protocols across studies complicates direct comparisons and standardization. Furthermore, bioavailability challenges inherent to natural compounds like CUR and RSV-such as poor solubility and rapid metabolism-were not systematically addressed, which may limit therapeutic extrapolation. Finally, the scope of this study was restricted to four well-characterized nutraceuticals, precluding evaluation of other candidates. The lack of harmonized global regulatory standards for purity, dosage, and health claims further complicates translational applicability. Future studies incorporating longitudinal human trials, bioavailability-optimized formulations, and regulatory alignment are urgently needed to address these gaps.

## 7 Future research directions and regulatory considerations

While this review underscores the therapeutic promise of nutraceuticals, significant gaps remain in translating preclinical findings into safe, evidence-based clinical practice. To enhance the robustness of future research, large-scale, longitudinal randomized controlled trials (RCTs) should be conducted to systematically evaluate the long-term safety and dose–response relationships of key nutraceuticals. Special emphasis should be placed on studying these compounds in vulnerable populations, including the elderly and individuals undergoing polypharmacy.

Additionally, there is a critical need to prioritize clinical trials that investigate nutraceutical-drug interactions, particularly combinations like QUE with chemotherapeutic agents or RSV with anticoagulants. Such studies would facilitate the development of risk-stratified guidelines, enabling clinicians to better predict and manage potential interactions in real-world settings.

Establishing centralized databases, similar to the WHO-Adverse Drug Reaction (ADR) database, for reporting nutraceutical-related adverse events could significantly improve real-world risk monitoring. These systems would facilitate the collection and analysis of post-market data, enabling researchers and regulators to better understand the safety profiles of commonly used nutraceuticals and guide evidence-based decision-making.

Advance research on nanotechnology-driven delivery systems, such as nanoemulsions and lipid-based carriers, to optimize the bioavailability of nutraceuticals and pharmaceuticals while mitigating potential toxicity risks. Exploring these innovative approaches can lead to more efficient and safer therapeutic interventions in the future.

## 8 Conclusion

Nutraceuticals, such as RSV, CUR, PPR, and QUE, hold significant promise for improving health and preventing disease due to their antioxidant, anti-inflammatory, and therapeutic properties. However, their “double-edged sword” nature-characterized by both protective benefits and potential risks-underscores the need for a cautious and balanced approach to their use. While these bioactive compounds offer numerous health benefits at low doses, high doses or prolonged use can lead to adverse effects, including organ-specific toxicity, pro-oxidant activity, and drug interactions. To ensure the safe and effective use of nutraceuticals, comprehensive toxicological assessments, including studies on toxicodynamics, genotoxicity, and drug interactions, are essential. Furthermore, advancements in delivery systems, such as nanotechnology-based formulations, offer promising solutions to enhance bioavailability while minimizing toxicity.

In summary, while nutraceuticals represent a valuable tool in promoting health and preventing disease, their full potential can only be realized through rigorous scientific research, personalized dosing strategies, and a comprehensive understanding of both their benefits and potential risks. The integration of nanotechnology and smart materials has emerged as a transformative approach to enhance the efficacy and safety of nutraceuticals. These innovations enable controlled release, improved bioavailability, and targeted delivery, thereby optimizing therapeutic outcomes while minimizing adverse effects. For instance, nanoemulsions and emulsomes have been shown to significantly enhance the absorption and stability of compounds like CUR and PPR, making them more effective for applications ranging from anti-inflammatory treatments to cancer therapy.
